# From promise to practice: artificial intelligence in mental health care in the MENA region

**DOI:** 10.3389/fpsyt.2026.1878052

**Published:** 2026-07-15

**Authors:** Sara El Hajj, Ahmad Nsouli, Mohamad Wehbe, George Saad, Fadi T. Maalouf

**Affiliations:** 1 Department of Psychiatry, American University of Beirut Medical Center, Beirut, Lebanon; 2 Faculty of Medicine, American University of Beirut, Beirut, Lebanon

**Keywords:** artificial intelligence, chatbots, conversational AI, digital mental health, MENA region

## Abstract

Mental health disorders represent a growing burden across the Middle East and North Africa (MENA) region, where depression and anxiety are highly prevalent amid conflict, displacement, and socioeconomic strain, affecting up to 40 percent of adults, yet treatment gaps remain at 80-95% due to provider shortages, financial strain, and cultural barriers. In this context, artificial intelligence (AI), in the form of large language models (LLMs) and specialized psychotherapy chatbots, may offer a scalable adjunct to help address these gaps through anonymous screening, predictive risk modeling, psychoeducation, and brief interventions. This narrative review examines current evidence of AI-driven conversational tools in mental health with a specific focus on their application, acceptance, and limitations within the MENA region. To do so, A structured search of MEDLINE and Embase (2000–2026) identified studies on conversational AI in mental health, prioritizing evidence from the MENA region and supplemented by relevant global literature. Overall, findings suggest that while these tools offer high accessibility and user engagement, particularly for low-intensity support, their effectiveness is limited by linguistic and cultural mismatches, including Arabic diglossia and poor alignment with locally grounded expressions of distress. At the same time, user acceptance reflects a paradox in which stigma and privacy concerns drive reliance on anonymous AI tools while simultaneously limiting trust in their clinical reliability, reinforcing a preference for hybrid models with human oversight. Taken together, these findings indicate that current systems remain insufficiently adapted to the MENA context, underscoring the need for culturally grounded, dialect-sensitive, and clinically supervised approaches to ensure safe and effective integration.

## Background

Mental health disorders remain a leading cause of disability worldwide, affecting over one billion people and contributing substantially to the global burden of disease (1, 2). Recent estimates indicate that mental health conditions account for approximately 14% of the global disease burden, with a substantial proportion emerging early in life and predicting chronic trajectories with long-term social, educational, and occupational impairment (3). Despite the high burden, access to care access to care remains markedly uneven across settings. It is estimated that only a minority of individuals with mental health conditions receive appropriate treatment, with treatment coverage reaching approximately 33% in high-income countries compared to as low as 5–10% in low- and middle-income countries (2, 4). As a result, the global treatment gap ranges from approximately 35–50% in high-income settings to over 75–90% in low-resource contexts. These inequities are particularly concerning among children and adolescents, in whom mental disorders frequently emerge but remain under-recognized and undertreated. In Lebanon, the national Psychopathology in Children and Adolescents in Lebanon Study (PALS) found that 32.7% of children and adolescents screened positive for a psychiatric disorder, yet only 5% of those affected had sought professional help (5). A more recent analysis from the same dataset further demonstrated that barriers to care were largely structural, with cost and lack of service availability identified as the most prominent obstacles to help-seeking (6).

This substantial and persistent treatment gap has prompted increasing interest in innovative approaches that can expand access to mental health care and complement existing services. Among these, artificial intelligence (AI) has emerged as a promising tool because of its potential to deliver scalable, accessible, and cost-effective mental health support. AI encompasses technologies capable of performing tasks that typically require human intelligence, while machine learning (ML) enables systems to identify patterns and generate predictions from large datasets. Recent advances in natural language processing (NLP), a branch of AI, have led to the development of large language models (LLMs), which can generate human-like responses and engage in complex conversations. These developments have facilitated the emergence of conversational AI tools, including mental health chatbots and virtual assistants, that can support psychoeducation, symptom monitoring, screening, and low-intensity psychological interventions.

Among the various AI applications in healthcare, conversational AI systems have emerged as particularly promising tools for mental health care. These technologies may offer key advantages, including anonymity, continuous availability, scalability, and convenience, which may help address stigma-related and structural barriers to care, particularly in underserved regions such as MENA (7–11).

A key distinction in the current landscape of AI in mental health lies between general-purpose large language models (LLMs) and purpose-built psychotherapy chatbots. LLMs, such as ChatGPT, are designed for broad conversational use and are capable of generating flexible, context-sensitive, and human-like responses, which may support psychoeducation, symptom monitoring, and early identification of psychological distress. In contrast, psychotherapy chatbots are typically built on structured therapeutic frameworks, such as cognitive behavioral therapy, and rely on predefined dialogue flows or rule-based systems that prioritize consistency and adherence to clinical principles (12). In addition, AI systems generate probabilistic outputs and may produce inaccurate or misleading information, particularly in emotionally complex or high-risk situations, underscoring the importance of clinical oversight and responsible implementation. Concerns have also emerged regarding the potential psychological effects of AI use itself, with recent evidence suggesting that frequent generative AI use may be associated with higher levels of depressive symptoms, possibly reflecting emotional reliance or reduced real-world social engagement. These findings highlight the need for longitudinal research, ethical monitoring frameworks similar to pharmacovigilance systems, and the incorporation of design safeguards, such as reality-testing prompts, to reduce potential harm and support safe user interaction. Given these opportunities and risks, AI is best conceptualized as a complementary tool that can enhance access, screening, and support, while preserving the essential role of human clinicians in delivering safe, culturally appropriate, and effective mental care (13).

Despite the potential advantages of AI in expanding access to mental health support, there are emerging real-world reports illustrating significant psychological harms associated with extensive engagement with large language model chatbots. In a detailed case report, a 26-year-old woman with no prior history of psychosis developed persistent delusional beliefs that she was communicating with her deceased brother through an AI chatbot; the chatbot repeatedly validated and reinforced these delusions, and her symptoms resolved only after hospitalization and antipsychotic treatment, though they recurred when she resumed immersive use of technology (14). Other documented cases describe individuals who, through prolonged interactions with ChatGPT and similar models, developed severe delusions, messianic thinking, romantic attachment to AI entities, or emotional dependency that contributed to interpersonal conflict, job loss, psychiatric hospitalization, and even suicide attempts (15). Although these reports do not establish causation and “AI psychosis” has not been recognized as an official psychiatric diagnosis, they highlight how the inherently agreeable and reinforcing nature of current AI chatbots can amplify distorted beliefs in vulnerable users (15). Importantly, these troubling incidents have occurred alongside widespread use of AI tools, by teenagers, adolescents, and adults alike, seeking mental health support, companionship, or self-help outside of formal clinical care, reflecting the ongoing tensions between the appeal of AI as accessible psychoeducational support and its ethical and safety risks when used without professional oversight (14).

Understanding user attitudes and acceptance is therefore critical for effective implementation. Evidence from diverse populations suggests that engagement depends on perceived usefulness, trust, and prior experience with digital tools. Young adults, particularly university students, are both early adopters of digital technologies and a population at elevated risk for untreated mental health problems. Studies indicate a general openness to AI for psychoeducation, symptom monitoring, and early risk identification, highlighting their potential to expand access to care and facilitate timely support. However, concerns remain regarding privacy, data security, and the lack of emotional understanding and contextual judgment in AI systems. Importantly, AI tools generate probabilistic outputs and may produce inaccurate or misleading responses, raising safety concerns, particularly in high-risk situations such as crisis management. Additionally, there is a concern that excessive reliance on AI could reduce engagement with human clinicians and potentially affect care quality if used as a substitute rather than a supplement to professional care. Hybrid care models, in which AI tools support screening, monitoring, and psychoeducation while preserving clinical oversight and therapeutic relationships, are therefore considered the most appropriate and effective implementation strategy (16–18).

Despite growing interest in the use of artificial intelligence in psychotherapy, several key gaps continue to limit its understanding and implementation. First, existing research has predominantly focused on clinical outcomes, particularly symptom reduction, with comparatively limited attention to users’ attitudes, experiences, and engagement within therapeutic contexts; this narrow focus restricts insight into how individuals interact with AI systems and whether these tools are acceptable or meaningful in real-world use.

Second, studies examining attitudes toward AI are often methodologically limited and frequently rely on brief or non-validated instruments, which reduces the reliability, comparability, and generalizability of findings (16). The absence of standardized and validated measures remains a major barrier to building a coherent and cumulative evidence base.

Third, cultural and contextual factors remain insufficiently examined, particularly in non-Western settings. Key dimensions such as trust, stigma, and comfort in discussing sensitive topics are likely to vary across sociocultural contexts but are rarely explored in a systematic manner. This limitation is especially pronounced in Arabic-speaking populations, where linguistic complexity and sociocultural norms may significantly shape user interaction with AI systems. Although emerging evidence from countries such as Turkey and Saudi Arabia suggests moderate openness to AI-assisted care, these findings remain limited in scope and highlight the need for region-specific, culturally grounded research in the broader MENA context (19).

Addressing these gaps is essential for the responsible integration of AI into mental health care. The development and use of validated, culturally adapted tools, such as an Arabic version of the Attitudes toward Artificial Intelligence in Psychotherapy Scale, would enable more rigorous assessment of user perceptions and facilitate meaningful cross-cultural comparisons (20). Such insights are critical for informing implementation strategies, particularly within hybrid care models, where AI supports psychoeducation and monitoring while clinicians provide relational and higher-level therapeutic interventions.

Accordingly, this review aims to synthesize current evidence on conversational AI in mental health care within the MENA region. It focuses on three domains: clinical and functional applications, user attitudes and acceptance among adolescents and young adults, and safety and ethical considerations, including potential psychological risks. By integrating findings across these areas, this review seeks to identify critical gaps and to inform culturally appropriate and clinically safe implementation of AI in mental health care.

## Methodology

### Search strategy and study selection

**Figure 1:**
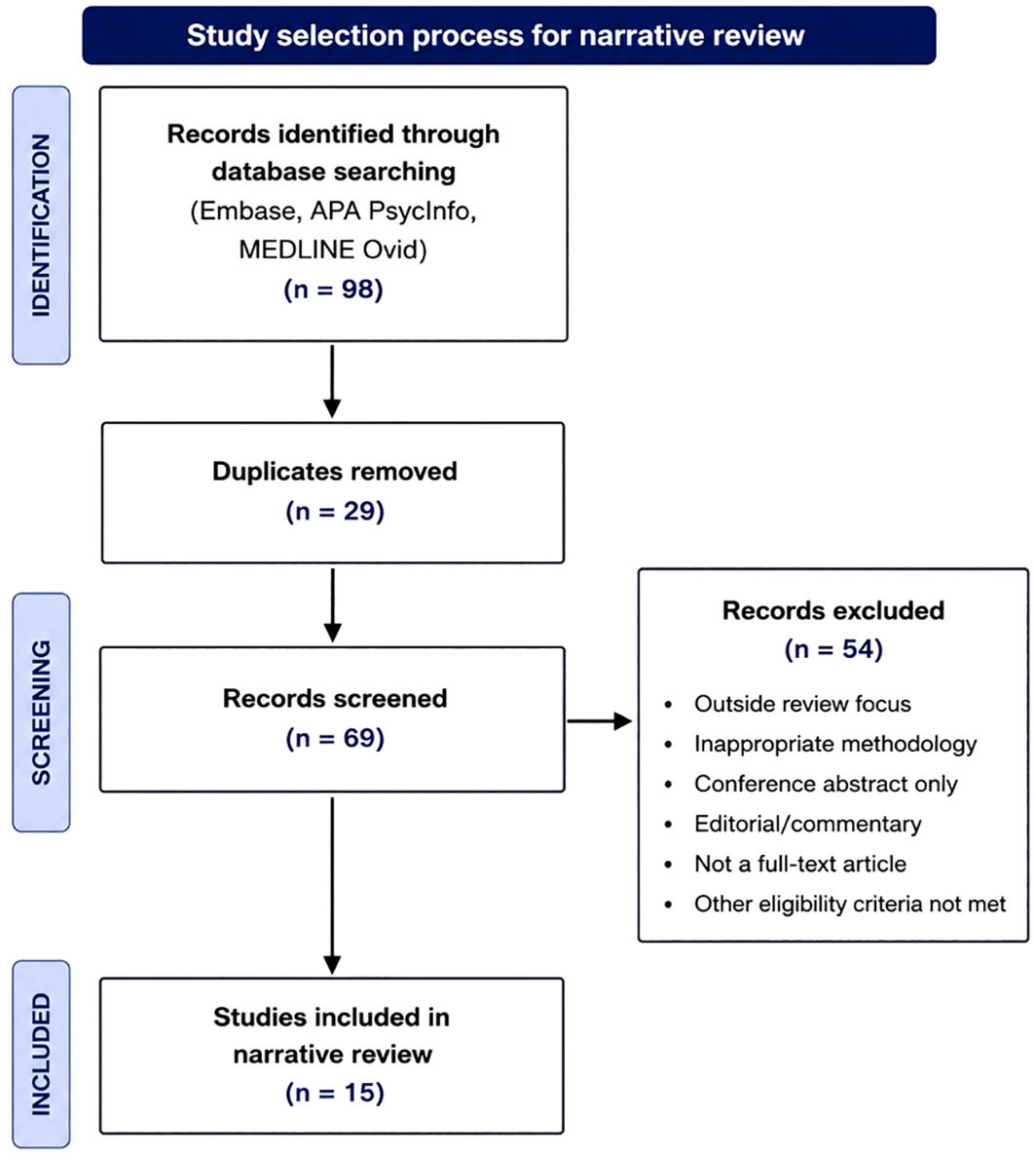
PRISMA flow diagram illustrating the study selection process for the narrative review. A total of 98 records were identified through searches of Embase, APA PsycInfo, and MEDLINE (Ovid). After removal of 29 duplicates, 69 records were screened. Fifty-four records were excluded for reasons shown. Fifteen studies were included in the final narrative review.

A structured but non-systematic search strategy was employed to identify relevant literature on artificial intelligence-driven conversational tools in mental health. Electronic including MEDLINE (Ovid), Embase, and APA Psychinfo were systematically searched for studies published between 2000 and 2026 (**Figure 1**).

Search terms were developed using combinations of three core concepts: artificial intelligence, mental health, and the Middle East and North Africa (MENA) region.

Artificial intelligence-related terms included “artificial intelligence,” “machine learning,” “natural language processing,” “generative artificial intelligence,” “large language model*,” “LLM*,” “chatbot*,” and “conversational agent*.”

Mental health–related terms included “mental health,” “mental disorders,” “depression,” “anxiety disorders,” “suicide,” “suicide prevention,” “psychiatry,” “child psychiatry,” “adolescent psychiatry,” “geriatric psychiatry,” “psychotherapy,” “group psychotherapy,” “interpersonal psychotherapy,” “cognitive behavioral therapy,” “dialectical behavior therapy,” “behavior therapy,” “mindfulness,” “mindfulness-based cognitive therapy,” “mindfulness-based stress reduction,” “acceptance and commitment therapy,” “exposure therapy,” “psychodynamic therapy,” “systematic desensitization,” “eye movement desensitization and reprocessing,” “treatment outcome,” “prolonged exposure therapy,” “substance-related disorders,” “personality disorder”, “ADHD”, and Neurodevelopmental Disorder”

The MENA concept included both regional identifiers (e.g., “Middle East,” “Africa, Northern,” “MENA region”) and individual country names (e.g., Algeria, Bahrain, Egypt, Iraq, Jordan, Kuwait, Lebanon, Libya, Morocco, Oman, Palestine, Qatar, Saudi Arabia, Syria, Tunisia, United Arab Emirates, and Yemen).

Combining all three concepts in all three data bases yielded 98 studies, after removing duplicated we were left with 69 articles that were further screened for eligibility. When the geographic MENA component was removed, the search yielded around 7,000 studies, reflecting the broader global literature in this field.

Studies were included if they (1) involved artificial intelligence–driven conversational tools (e.g., chatbots, conversational agents, or large language model–based systems), and (2) addressed mental health, psychiatric care, or psychological support. Priority was given to studies conducted in or directly relevant to the MENA region. However, given the emerging and rapidly evolving nature of artificial intelligence in mental health, some studies conducted outside the region were also included when they provided relevant insights into technological development, implementation strategies, clinical applications, or ethical considerations. These studies were used to contextualize and supplement the limited regional evidence base.

Studies were excluded if they focused on non-conversational artificial intelligence applications or did not address mental health outcomes or applications.

Given the narrative and exploratory nature of this review, studies were not excluded based on methodological design or formal quality appraisal. Included articles were reviewed in full and interpreted qualitatively. The aim was not to exhaustively capture all available studies, but rather to identify key patterns, emerging themes, and conceptual insights relevant to the use of conversational artificial intelligence in mental health, particularly within the MENA context.

### Search strategy and study selection

In this review, the term “digital interventions” and “AI conversational tools” were used to refer to systems that simulate human-like interaction through text or speech, including chatbots, conversational agents, and large language model-based systems.

The term “mental health” is used broadly to encompass psychiatric disorders, psychological distress, and the delivery of mental health care, including psychotherapy and supportive interventions.

### Data extraction

Data were extracted using a structured approach to ensure consistency across included studies. Extracted variables included study characteristics (author, year, and country), study design and population, type of artificial intelligence tool, mental health domain addressed, and key findings and reported outcomes. This approach enabled systematic comparison of studies while capturing both clinical and technological dimensions of the interventions.

### Synthesis approach

Given the heterogeneity of study designs, populations, and artificial intelligence technologies, a quantitative synthesis was not appropriate; therefore, a structured narrative synthesis was conducted using a predefined thematic framework aligned with the objectives of the review. Studies were systematically categorized into key domains, including clinical applications, effectiveness and reported outcomes, user engagement and acceptance, and implementation considerations such as feasibility, cultural adaptation, and ethical challenges. Within each domain, findings were compared across studies to identify convergent patterns, inconsistencies, and areas of limited evidence.

## Main body

### AI technologies in mental health

Artificial intelligence (AI) technologies, including general-purpose large language models (LLMs) and specialized psychotherapy chatbots, are increasingly being integrated into mental health care to expand its reach and effectiveness (21–23). These tools offer scalable and accessible support, particularly in underserved regions where traditional services may be limited. By providing anonymous and low cost early interventions and maintaining high levels of user engagement, AI driven solutions have the potential to bridge gaps in care and extend mental health support to populations that face significant barriers to conventional treatment (16, 24).

A key distinction is necessary to make sense of the current landscape of AI use in mental health, particularly as systems with very different purposes are often treated as interchangeable. General-purpose large language models are designed for broad conversational and task-oriented applications, yet they are increasingly used in ways that resemble therapeutic support (Torous & Blease, 2024. Although these models may draw on extensive clinically relevant knowledge, this does not guarantee that their responses will be safe, consistent, or appropriate in mental health contexts, where careful boundaries and reliability are essential (25).

In contrast, purpose-built psychotherapy chatbots are developed with more narrowly defined clinical goals, such as delivering cognitive behavioral therapy (CBT) techniques, providing psychoeducation, or supporting guided self-help (13, 26). Their design typically relies on structured dialogue flows and clearly defined operational limits, which shape how they respond and make their behavior more predictable. Some of these systems are supported by empirical evidence, although this evidence is often limited to short-term outcomes and specific populations rather than broader clinical effectiveness (21).

The distinction ultimately reflects a deeper trade-off between flexibility and control. Generative systems are capable of producing fluid, context-sensitive, and often empathic responses, allowing for a high degree of personalization and user engagement that can resemble aspects of a therapeutic alliance. Purpose-built systems, by comparison, place greater emphasis on safety, consistency, and adherence to established therapeutic protocols, often using rule-based or retrieval-driven approaches to deliver structured interventions such as CBT (13, 27). Emerging research, including work in Arabic natural language processing within the MENA region, increasingly points toward hybrid approaches that seek to combine the adaptability of generative models with the safeguards and clinical grounding of structured systems (28).

### User attitudes and acceptance of AI in mental health care

Across recent systematic reviews and meta-analyses, attitudes toward AI in mental health care are cautiously optimistic, shaped by usefulness, accessibility, therapeutic alliance, and trust. A meta-analysis of 35 studies reported moderate reductions in depression and distress, with user satisfaction driven by empathic, non-judgmental communication, while poor understanding and impersonality reduced engagement (22). A broader review also found strong engagement with chatbot platforms like Wysa, where greater usage correlated with larger symptom improvements and users valued anonymity, 24/7 availability, and personalized feedback (29).

Acceptance increases when AI is transparent, explainable, and integrated into clinical workflows, though data imbalance, privacy risks, and limited interpretability remain barriers (30). Individual factors also matter: higher agreeableness, digital competence, and AI awareness predict greater perceived usefulness, while distress strengthens the link between attitudes and actual use (31) (**Table 1**). Preferences vary; more than half of highly educated Turkish participants favored AI therapy for discussing sensitive topics and accessibility, but concerns about empathy and data security persisted (16).

**Table 1:** AI-related regulatory developments across MENA countries.

Country	AI strategy/regulatory initiative	Data protection framework	Relevance to mental health AI
United Arab Emirates	UAE National AI Strategy 2031	Federal Personal Data Protection Law (PDPL)	Provides national framework for responsible AI deployment; mental-health-specific guidance remains limited
Saudi Arabia	Saudi Data and Artificial Intelligence Authority (SDAIA); National Strategy for Data & AI	Personal Data Protection Law (PDPL)	Supports AI innovation and governance; relevant to clinical AI and digital health platforms
Qatar	National Artificial Intelligence Strategy	Personal Data Privacy Protection Law No.13 of 2016	Encourages AI integration across sectors including healthcare
Bahrain	National AI initiatives under Economic Vision 2030	Personal Data Protection Law No.30 of 2018	Early regulatory environment supporting AI adoption
Oman	National Digital Economy Program	Personal Data Protection Law (2022)	Emerging governance structures relevant to healthcare AI
Egypt	National AI Strategy	Personal Data Protection Law No.151 (2020)	Growing interest in AI-enabled healthcare and digital transformation
Jordan	National AI Policy initiatives	Personal Data Protection legislation under development	Limited mental-health-specific AI governance
Lebanon/Remaining MENA countries	No dedicated national AI strategy currently implemented	Fragmented data protection framework	Lack of clear governance may create challenges for clinical implementation of AI mental health tools

Despite increasing governmental investment in AI and digital transformation across several MENA countries, none currently possess regulatory frameworks specifically designed for conversational AI in mental healthcare. Existing policies largely focus on innovation, data governance, and digital transformation rather than clinical oversight, safety monitoring, or accountability mechanisms for AI-enabled mental health interventions.

Clinicians reflect a similar balance: 70% anticipate documentation efficiency gains, 75% expect patients to consult AI before care, and 90% call for more training, while concerns about bias, privacy, and therapeutic impact remain (23). Overall, acceptance is strong when AI demonstrates effectiveness, personalization, and privacy protection, but sustained adoption depends on addressing ethical, transparency, and training challenges.

In MENA contexts, the application of trustworthy AI in mental health is shaped by regulatory fragmentation, linguistic diversity, and strong sociocultural sensitivities around privacy and stigma (**Table 1**). These factors influence how core attributes such as accountability, competence, and fairness are experienced in practice. Surveys conducted in Turkey and Saudi Arabia report moderate to high willingness to engage with AI-assisted psychotherapy, particularly when tools are framed as supportive rather than substitutive of human care (16, 20). In contrast, studies from the United States and Australia reveal substantially lower adoption rates, with users expressing skepticism regarding effectiveness and a preference for human therapists despite acknowledging the potential benefits of AI for reducing cost, stigma, and waiting times (17, 24).

A recurring theme across studies is the appeal of anonymity and nonjudgmental interaction, especially among young adults who may avoid traditional psychotherapy due to stigma or fear of disclosure. University students, in particular, demonstrate cautious interest in AI tools for psychoeducation, mood tracking, and brief coping strategies while remaining reluctant to rely on them for emotionally complex or crisis-related care (16, 17). Clinical and feasibility studies of chatbots such as Woebot and Wysa report acceptable engagement and perceived helpfulness, with users valuing ease of use and immediacy (13, 26). However, acceptance tends to decline when AI tools are perceived as lacking emotional depth or contextual understanding, underscoring the importance of received empathy and therapeutic alliance in user engagement (16, 32).

Trust represents a central determinant of acceptance. Users frequently express concerns regarding data privacy, confidentiality, and secondary use of personal information (16, 24, 33). Mental health professionals echo this ambivalence, recognizing AI’s potential for screening, psychoeducation, and symptom monitoring, while questioning its capacity to support nuanced clinical judgement or relational care (24, 34). Notably, both users and clinicians tend to favor hybrid models, in which AI tools augment rather than replace human clinicians, suggesting that acceptance increases when AI is positioned as a complementary support within existing care pathways (28, 34).

Accountability remains a central concern, particularly for general-purpose LLMs deployed through international platforms that operate outside national healthcare governance structures, leaving responsibility for system behavior, incident response, and user harm unclear (35). Even where purpose-built chatbots are embedded within local initiatives, users often remain uncertain about who governs these systems or how risks are managed (36). This lack of clarity is compounded by the way users engage with AI, as stigma surrounding mental health can encourage reliance on anonymous, non-judgemental support. The conversational fluency of LLMs can foster relationship-like interactions that increase engagement, but also raise the risk of over-trust, misinterpretation, and confusion about the system’s role or capabilities (27, 33).

These challenges are further intensified by limitations in competence, fairness, and transparency. Performance in Arabic remains inconsistent, particularly across dialects and culturally specific expressions of distress, reflecting gaps in datasets and evaluation frameworks (37, 38). At the same time, both general-purpose and purpose-built systems often rely on therapeutic models that are not fully adapted to local cultural contexts, including religious and family-oriented understandings of mental health. Concerns about equity and relevance are further amplified by the under-representation of Arabic language and cultural perspectives in training data and natural language processing pipelines (27, 39).

Issues of transparency further complicate trust. Users may interpret fluent and coherent outputs as evidence of expertise, while simultaneously reporting uncertainty about system capabilities, training data, and limitations (24, 27). This creates a dual dynamic of over-trust and skepticism, shaped in part by varying levels of digital literacy and familiarity with artificial intelligence systems.

Overall, attitudes toward artificial intelligence in mental health are neither uniformly optimistic nor dismissive. Rather, acceptance appears contingent upon cultural context, perceived usefulness, trust in data handling, and clarity regarding the role of AI within care delivery (16, 24, 34). These findings underscore the need for culturally sensitive assessment tools and transparent implementation strategies, particularly in under-researched regions such as the Middle East and North Africa, where structural, linguistic, and sociocultural factors uniquely shape engagement with AI-enabled mental health interventions (40).

Despite growing interest in user and clinician attitudes toward AI-assisted mental health care, the current evidence base remains subject to several important methodological limitations. Much of the literature relies on cross-sectional survey designs, convenience sampling, and self-reported measures, limiting causal inference and generalizability. Furthermore, most studies originate from North America, Europe, or other high-income settings, while evidence from Arabic-speaking populations and the broader MENA region remains scarce. Several investigations use newly developed or non-validated instruments to assess attitudes toward AI, making comparisons across studies difficult and limiting confidence in reported findings. Sample sizes are often modest, and few studies evaluate actual behavioral use or long-term engagement with AI systems in routine mental health care. These limitations are particularly important in the MENA region, where linguistic diversity, cultural norms, stigma, and differing healthcare infrastructures may substantially influence acceptance and implementation. Future research should prioritize longitudinal, culturally adapted, and methodologically rigorous studies that can better inform the safe and effective integration of AI into mental health services.

### Stigma and privacy

In the MENA region, the integration of AI into mental healthcare is driven by a profound “paradox of access.” While the prevalence of mental health disorders is estimated to range from 11% to over 40%, the journey toward traditional clinical care is often obstructed by deeply rooted socio-cultural stigmas (41). Stigma and shame continue to shape perceptions of mental health in the Arab region, despite growing awareness and increasing acceptance driven by public health education and awareness campaigns (42). In many conservative societies, mental health services remain underutilized due to negative attitudes toward them (43). Instead, many individuals in Arab communities tend to seek informal support, such as religious advisors, close friends, or family members, rather than formal mental health services in an effort to avoid the stigma associated with seeking professional psychiatric care (44). When it comes to using digital technology for mental health in the MENA region, cultural and religious aspects play an important role in the acceptance and adoption of digital health interventions. Recently, many Arab individuals have preferred digital mental health interventions over traditional in-person consultations (45). This environment, characterized by persistent stigma alongside growing openness to digital technologies, creates a unique context that may facilitate the integration of AI into mental health services.

The pervasive nature of mental health stigma in many MENA societies serves as a primary lens through which AI interventions are perceived. Traditional pathways to care are often obstructed by the fear of social “shame” (*ayb*) or the potential for reputational damage to the family unit (46). In this context, AI-driven platforms offer a unique “buffer” that human clinicians cannot; the perceived anonymity of a digital interface may lower the threshold for help-seeking among populations who would otherwise avoid psychiatric service (47) (**Table 2**).

**Table 2:** Summary of studies included in the synthesis of this review.

Author (year)	Country	Study design	Population	AI tool/technology	Mental health domain	Key findings	Limitations
Abd Alrazaq et al. (21)	Multiple countries	Systematic review & meta-analysis	General population	Mental health chatbots	Anxiety, Depression, Psychological distress	Chatbots demonstrated effectiveness and acceptable safety profile	Heterogeneity of interventions
Alanzi et al. (19)	Saudi Arabia	Cross-sectional survey	Generations X, Y, Z	AI virtual assistants	Mental healthcare acceptance	Moderate to high acceptance driven by perceived usefulness and trust	Self-reported attitudes only
Mamdouh et al. (45)	Egypt	Cross-sectional survey	University students	Digital mental health tools	Mental healthcare utilization	Students generally open to digital mental healthcare	Did not specifically evaluate conversational AI
Mina et al. (48)	Lebanon	Cross-sectional study	Young women	AI chatbots	Sensitive health support	Privacy and reduced embarrassment increased engagement	Non-clinical population
Abdelwahed et al. (49)	21 Arab countries	Multinational survey	General population	AI healthcare chatbots	Healthcare assistance and mental health support	Psychological distress associated with increased AI chatbot use	Cross-sectional design
Sharif et al. (50)	MENA	Cross-sectional survey	Mental health professionals	AI mental health tools	Professional attitudes	Clinicians cautiously optimistic but concerned about ethics and safety	Limited regional representation
Al-Ansari & Al-Medfa (51)	Bahrain	Survey	Psychiatrists	AI systems in psychiatry	Clinical applications	Positive attitudes toward structured tasks; skepticism regarding psychotherapy	Small sample
Al-Otaibi et al. (52)	Saudi Arabia	Evaluation study	Arabic-language interactions	ChatGPT	Arabic mental health support	Significant communication and contextual errors identified	Single platform evaluation

There is limited empirical data to back the rapid deployment of AI-driven mental health tools in the MENA region. Consequently, understanding the potential impact of these technologies requires a comparative analysis of other collectivist cultures where the friction between individualistic digital interventions and communal healing traditions is better documented. Research in mental health literacy consistently shows that in collectivist societies such as India, help-seeking is strongly shaped by family, friends, and the broader community, which are central to the healing process, whereas these influences are often far less prominent in European and American samples (53). This distinction is particularly important in the MENA context, where the growing shift toward individualistic, self-guided digital tools may conflict with deeply rooted beliefs that frame healing as a communal rather than an individual process. Therefore, the preference for digital interaction through a chatbot is paradoxical. While the “judgement-free” nature of chatbots can be appealing, it also simultaneously risks reinforcing the idea that mental health is a secret to be kept, rather than a medical condition to be integrated into social support structures, which is essential for effective psychiatric care in the Arab region.

Despite all this, evidence has suggested that for many, the perceived safety of an anonymous digital interaction may currently outweigh the necessity for clinical perfection (48). For instance, among young women in Lebanon, 43.4% cited the avoidance of embarrassment and 33.3% cited a fear of judgment as primary motivators for engaging with AI chatbots for sensitive health concerns (48). This trend is reflected broadly across 21 Arab countries, where individuals experiencing psychological distress show significantly higher odds of using AI, suggesting that for the most vulnerable, the chatbot represents a vital, low-friction entry point into a healthcare system that otherwise feels inaccessible (49) (**Table 2**).

### Dialectal complexity and NLP constraints

The efficacy of AI-driven mental health tools in the MENA region is deeply constrained by the linguistic and cultural complexity of the Arabic language. Most Natural Language Processing (NLP) systems are trained predominantly on Modern Standard Arabic (MSA), whereas real-world communication occurs largely in spoken Arabic (SA), a set of diverse regional dialects that differ substantially in lexicon, syntax, and morphology (54). This diglossic structure creates a mismatch between training data and real-world deployment, with dialectal variation demonstrably reducing model performance and classification accuracy (55). Empirical findings reinforce this gap as semantic textual similarity models achieve lower performance on dialects such as Egyptian (77.5%) and Saudi Arabic (76%) compared to MSA (81%), highlighting the limitations of current models in processing everyday language (56).

While digital literacy is relatively high in parts of the MENA region, most conversational agents rely on MSA, a formal register that lacks the emotional nuance and immediacy required for therapeutic engagement (37). In practice, individuals express psychological distress in dialect and through culturally embedded expressions, which many AI systems are not equipped to interpret accurately (57). Indeed, evaluations of models such as ChatGPT-3.5 in Arabic mental health contexts have identified extensive communication errors, including grammatical inconsistencies and a lack of culturally appropriate empathic cues (52).

Beyond dialect, the expression of distress in Arab populations is often mediated through somatic metaphors and culturally specific “idioms of distress” (52). The DSM-5-TR recognizes that such idioms are not incidental but central to how individuals communicate suffering, shaping clinical presentations and patient–clinician interactions. Expressions such as “a weight on the chest,” “tightness in the heart,” or sensations of heat and pressure are common in many cultural contexts but may be misinterpreted if taken literally. In Arab populations, these metaphors are deeply embedded in linguistic and cultural frameworks, with evidence suggesting that symptom expression is closely tied to culturally specific explanatory models of illness (58). This is particularly critical in Arab contexts, where somatic symptom presentations are both prevalent and under-recognized, with reported rates ranging from 12% to 46% (59).

Addressing these challenges requires a shift toward deep cultural and linguistic localization. Current evidence suggests that the majority of culturally diverse users perceive digital mental health tools as insufficiently tailored, citing limited cultural representation and competency as key barriers (60). Effective AI systems must instead incorporate dialect-sensitive NLP, culturally grounded idioms of distress, and co-designed frameworks developed in collaboration with target populations (61). This need is further underscored by findings that cultural differences shape how users engage with digital tools, including variations in emotional expression and willingness to disclose sensitive information (62).

### Clinician perspectives and the limits of AI in psychiatry

Despite growing public engagement with AI driven mental health tools, professional acceptance in the MENA region remains cautious and selective (50). Clinicians show openness to AI in structured, analytical tasks such as diagnostic support and prognosis, yet continue to question its role in the relational core of psychiatric care (50, 63) (**Table 2**). Empathy, contextual judgment, and the nuanced interpretation of patient narratives are still widely regarded as inherently human capacities that current AI systems cannot replicate.

This skepticism is reflected in the persistent gap between awareness and implementation. Although familiarity with AI tools is high among healthcare professionals, actual clinical use remains limited, pointing to concerns about reliability, ethical responsibility, and institutional readiness. Similar doubts are echoed by the public, reinforcing the perception that AI, while useful, is not yet fully trustworthy in sensitive mental health contexts (49).

AI is increasingly positioned as an accessible and stigma free entry point into care, yet its long-term integration into formal mental health systems remains constrained by cultural, linguistic, and professional barriers. Moving forward, the role of AI will likely depend on its ability to complement rather than replace human care, embedding itself within culturally informed, clinically supervised frameworks. Without this alignment, AI risks remaining a peripheral solution, valued for its accessibility but limited in its therapeutic depth and sustainability within the region’s healthcare systems.

### AI governance and regulatory considerations in MENA

The rapid expansion of AI in healthcare has prompted several MENA countries to develop national AI strategies and data governance frameworks. The United Arab Emirates, Saudi Arabia, Qatar, Bahrain, Oman, and Egypt have introduced initiatives aimed at promoting responsible AI development, digital transformation, and data protection. However, few existing frameworks specifically address conversational AI applications in mental healthcare. Current policies largely focus on innovation and data governance rather than clinical accountability, safety monitoring, algorithmic transparency, and management of AI-related harms.

## Conclusion/take home messages

The Middle East and North Africa region faces a significant mental health treatment gap exacerbated by stigma, shortages in workforce, and structural barriers to care. Conversational AI-tools offer a scalable and anonymous means of expanding access through screening, psychoeducation, and low-intensity interventions. However, emerging evidence highlights important limitations, including inconsistent responses, lack of clinical safeguards, and the potential for psychological harms such as emotional overreliance and reinforcement of maladaptive beliefs when used without supervision.

Translating the promise of artificial intelligence into meaningful mental health improvements in the MENA region will require a coordinated effort across research, clinical practice, technology development, and policy. First, future research should prioritize the development and validation of culturally and linguistically adapted AI systems capable of recognizing regional dialects, culturally specific expressions of distress, and local help-seeking behaviors. Greater investment is also needed in longitudinal and real-world implementation studies, as much of the existing evidence relies on short-term outcomes and cross-sectional assessments.

Second, AI tools should be integrated within hybrid models of care rather than deployed as stand-alone therapeutic replacements. Such models may leverage AI for psychoeducation, symptom monitoring, screening, triage, and low-intensity support while preserving clinician oversight for diagnosis, risk assessment, and therapeutic decision-making. This approach may be particularly valuable in settings facing workforce shortages and long waiting times.

Third, developers should adopt participatory and co-design approaches involving patients, clinicians, linguists, ethicists, and community stakeholders throughout the development process. Such collaboration can help ensure that AI systems are culturally appropriate, clinically relevant, and responsive to the unique social and religious contexts of MENA populations.

Fourth, policymakers and regulatory bodies should establish clear governance frameworks addressing data protection, accountability, transparency, bias mitigation, and clinical safety. Given the sensitive nature of mental health information, robust safeguards are essential to maintain public trust and protect users from potential harms. Particular attention should be given to defining responsibilities when AI systems generate inaccurate, harmful, or clinically inappropriate recommendations.

Finally, investments in digital literacy and clinician training will be critical to support responsible implementation. Mental health professionals should be equipped to understand the capabilities and limitations of AI systems, while users should receive clear information regarding the role of these technologies, their limitations, and appropriate circumstances for seeking human support. Through culturally grounded development, appropriate regulation, and clinically supervised implementation, AI has the potential to become a valuable component of mental health care delivery in the MENA region while preserving the central role of human therapeutic relationships.
